# LPCAT4 Knockdown Alters Barrier Integrity and Cellular Bioenergetics in Human Urothelium

**DOI:** 10.3390/ijms231911871

**Published:** 2022-10-06

**Authors:** Andrew S. Mason, Claire L. Varley, Olivia M. Foody, Xiang Li, Katie Skinner, Dawn Walker, Tony R. Larson, Daisuke Wakamatsu, Simon C. Baker, Jennifer Southgate

**Affiliations:** 1 Jack Birch Unit for Molecular Carcinogenesis, Department of Biology and York Biomedical Research Institute, University of York, Heslington, York YO10 5DD, UK; 2 Department of Computer Science, University of Sheffield, Regent Court, 211 Portobello, Sheffield S1 4DP, UK; 3 Metabolomics and Proteomics Laboratory, Bioscience Technology Facility, The Department of Biology, The University of York, Heslington, York YO10 5DD, UK; 4 Pharmaceutical Co., Ltd., 3-1-1 Sakurai, Shimamoto-cho, Mishima-gun, Osaka 618-8585, Japan

**Keywords:** urothelium, transcriptomics, lipidomics, transepithelial electrical resistance, glycerophospholipids, phosphatidylcholine, wound healing, AGPAT7, LPEAT2, LPLAT10

## Abstract

Urothelium is a transitional, stratified epithelium that lines the lower urinary tract, providing a tight barrier to urine whilst retaining the capacity to stretch and rapidly resolve damage. The role of glycerophospholipids in urothelial barrier function is largely unknown, despite their importance in membrane structural integrity, protein complex assembly, and the master regulatory role of PPARγ in urothelial differentiation. We performed lipidomic and transcriptomic characterisation of urothelial differentiation, revealing a metabolic switch signature from fatty acid synthesis to lipid remodelling, including 5-fold upregulation of *LPCAT4*. *LPCAT4* knockdown urothelial cultures exhibited an impaired proliferation rate but developed elevated trans-epithelial electrical resistances upon differentiation, associated with a reduced and delayed capacity to restitute barrier function after wounding. Specific reduction in 18:1 PC fatty acyl chains upon knockdown was consistent with LPCAT4 specificity, but was unlikely to elicit broad barrier function changes. However, transcriptomic analysis of *LPCAT4* knockdown supported an LPC-induced reduction in DAG availability, predicted to limit PKC activity, and TSPO abundance, predicted to limit endogenous ATP. These phenotypes were confirmed by PKC and TSPO inhibition. Together, these data suggest an integral role for lipid mediators in urothelial barrier function and highlight the strength of combined lipidomic and transcriptomic analyses for characterising tissue homeostasis.

## 1. Introduction

Glycerophospholipids are the major constituent of biological membranes [1,2]. These amphipathic molecules consist of glycerol, two hydrophobic fatty acids, likely of variable length and saturation, and a hydrophilic phosphate ester. All glycerophospholipids are synthesised from glycerol-3-phosphate through the Kennedy Pathway [3,4,5], with the addition of different phosphate esters defining the major subgroups: phosphatidic acid (PA), phosphatidylcholine (PC), phosphatidylethanolamine (PE), phosphatidylinositol (PI), phosphatidylglycerol (PG), and phosphatidylserine (PS). PC is typically the most abundant glycerophospholipid in mammalian cell membranes, with abundance highly dependent on species, cell type and cellular compartment [1,6]. Glycerophospholipids are remodelled and diversified in membranes through the Lands’ Cycle, where deacylation by phospholipase A_2_ family members is followed by reacylation, incorporating new fatty acyls by acyltransferases with substrate specificity [7]. Such diversity is highly important for membrane thickness and fluidity, intrinsic curvature and lateral pressure profile. In addition to their structural role within membranes, glycerophospholipid derivatives, such as lysophospholipids, eicosanoids and diacylglycerol (DAG), are critical components of cell signalling [8].

Despite their importance to cellular homeostasis and physical properties, the glycerophospholipid environment remains poorly understood in many tissues.

The human urinary tract is lined by urothelium, a transitional epithelium that uses intercellular junctions anchored in cell membranes to form a highly specialised tight epithelial barrier to urine retained despite undergoing continuous physical changes from urine flow and storage. Urothelial differentiation is regulated by the nuclear receptor peroxisome proliferator-activated receptor gamma (PPARγ), well-recognised in lipid research as the master regulator of adipogenesis [9,10,11,12]. Despite this, little is known about human urothelial glycerophospholipid biology, even though both benign and malignant urothelial disease undergo disruption of barrier integrity and restitution [13,14,15], and urine has the potential to provide non-invasive diagnosis and monitoring [15,16,17], as long as the healthy state is fully understood.

Here, we exploited our ability to derive biomimetic, functional urothelium from in vitro-propagated normal human urothelial (NHU) cells derived from surgical samples [18,19] to perform the first joint transcriptomic and lipidomic characterisation of urothelial differentiation. Whilst the overall glycerophospholipid profile did not change significantly upon differentiation, Lysophosphatidylcholine acyltransferase 4 (*LPCAT4*) was significantly upregulated. Stable *LPCAT4* knockdown in NHU cells resulted in reduced proliferation and, after differentiation, a tighter epithelial barrier but impaired wound restitution ability. Specific transcriptomic and glycerophospholipid changes in the *LPCAT4* knockdowns supported a role for excess LPC to inhibit protein kinase C activity and alter barrier mechanics, and also, independently, limit the availability of intracellular ATP. Together, these results suggest that lipid mediators play a significant functional role in human urothelial homeostasis.

## 2. Results

### 2.1. Urothelial Differentiation Induces Glycerophospholipid Remodelling and Triglyceride Accumulation

**Figure 1:**
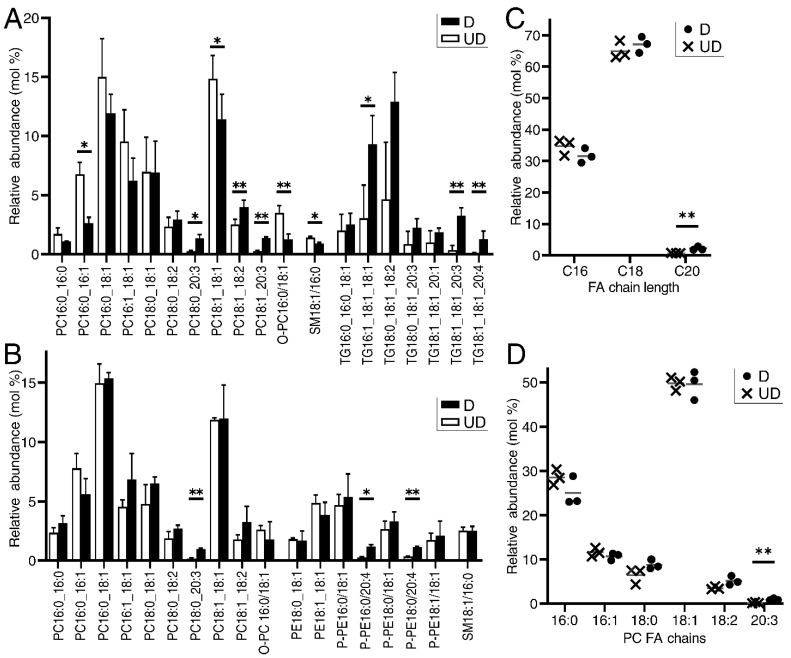
Lipidomic profiling of human urothelial cells in undifferentiated [UD] and in vitro differentiated [D] states. Plots built on LC-MS/MS profiles of lipid species with an average proportion ≥1% in either differentiation state, either for the positive ion (**A**) or negative ion (**B**) collections. In each plot, bars represent average relative proportion across 3 NHU donor backgrounds with statistical comparisons based on paired post hoc Tukey’s tests from an original 2-way ANOVA with BH-corrected significance values. (**C**) Glycerophospholipid fatty acyl chain length (using data from the negative ion collection only) increased significantly upon differentiation, with reductions in C16 proportions and elevations of both C18 and C20. (**D**) PC changes were limited upon differentiation, but these data highlight the dominant proportion of 18:1 fatty acyl chains. In (**C**,**D**), grey horizontal lines represent the average of 3 indicated NHU donor backgrounds. Significance values were generated by paired ‘*t*’ tests. Across all plots, ** indicates *p* < 0.01 and * indicates *p* < 0.05.

Twin cultures of undifferentiated and in vitro-redifferentiated human urothelial cells from three donor backgrounds were profiled by LC-MS (Liquid Chromatography-Mass Spectrometry)/MS. Lipid species with a relative abundance greater than 1% in at least one sample were manually annotated from both positive (Figure 1A) and negative ion modes (Figure 1B). A total of 12 distinct PC, plasmanyl-PC (O-PC) and sphingomyelin (SM) lipids were found in both modes, 7 triglycerides (TG) were identified in the positive mode alone, and 2 PE and 5 plasmenyl-PE species were identified only in the negative mode. There were no significant spectra consistent with PG, PI or PS glycerophospholipids identified in either mode, however these subgroups were detectable from the lipid standards.

Pooled TG species abundance increased by 279% upon differentiation (*p* = 0.048). Conversely, glycerophospholipid intensities were not, overall, differentiation-associated. Glycerophospholipid intensities in the negative mode were highly similar across samples (Figure 1B), and sample lipid profiles were strongly correlated within treatment groups (ρ = 0.945; *p* = 9.48 × 10^−12^). PC, O-PC and SM abundances also correlated well between modes (*n* = 6; average ρ = 0.913), reducing confidence in statistically significant PC abundance changes observed only in the positive mode analysis.

Whilst glycerophospholipid abundance was not greatly affected by differentiation, there was evidence of remodelling, with a significant increase in utilised chain length (*p* = 0.014; Figure 1C) and slight enrichment of the unsaturated fatty acyl chain proportion (*p* = 0.070), both driven by increased abundance of polyunsaturated C20 fatty acyls upon differentiation. PC was the most abundantly detected lipid class, with a stable PC:PE:SM ratio of ~24:8:1 across samples and differentiation states. PC molecules predominantly contained monounsaturated C18 fatty acyls (49.7%; sd 2.3%; Figure 1D).

### 2.2. LPCAT4 Identified as a Differentiation-Associated Remodeller of Phosphatidylcholine in Urothelium

**Figure 2:**
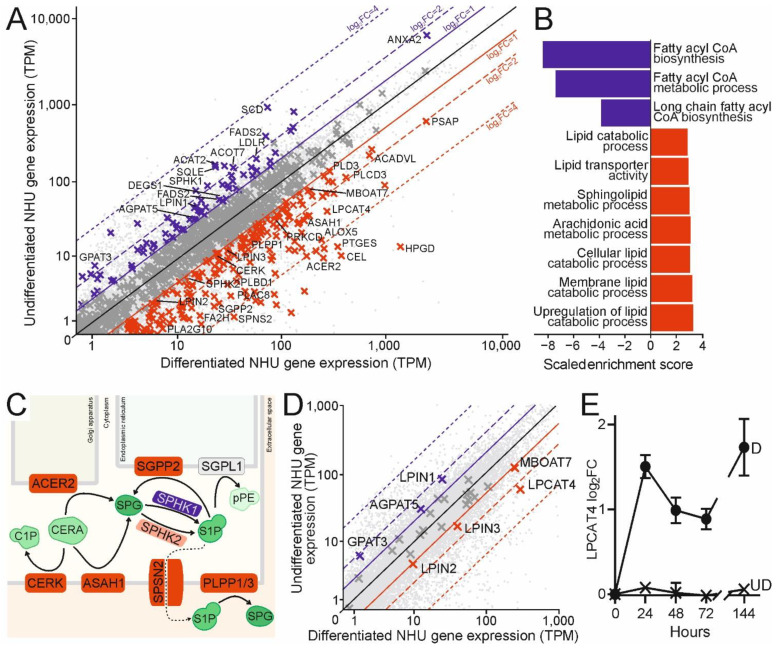
Modulation of human urothelial lipid biosynthesis induced upon differentiation. (**A**) Gene expression plot comparison of three donor-matched cultures before [UD] and after [D] induction of the NHU differentiation protocol. Genes with gene ontology terms linked to lipid processing are shown as crosses, otherwise dots. Red crosses exhibit greater than doubling of expression in the differentiated cultures, and vice versa for the blue crosses, when corrected *p* values were less than 0.05. Genes of particular interest are indicated. (**B**) Lipid pathways enriched in differentiated (red) or undifferentiated (blue) states. Scaled enrichment score is equal to the GSEA normalised enrichment score multiplied by the reciprocal of the FDR. (**C**) Urothelial “sphingolipid rheostat” showing the upregulation of gene transcript upon differentiation where protein products deplete ceramide (CERA) or sphingosine-1-phosphate (S1P), likely increasing sphingosine (SPG) levels. Upregulated genes in red, downregulated in blue, partial upregulation in pink, no significant change in grey. Changing genes are named in (**A**). Metabolites in green. C1P: ceramide-1-phosphate; pPE: phosphoethanolamine. ACER2 hydrolysis of ceramides commonly produces sphingosine and stearic acid (18:0); the latter may explain the slight increase in 18:0 PC fatty acyl chains seen upon differentiation (Figure 1D). (**D**) Same as the expression plot in (**A)** but highlighting genes from the Kennedy Pathway or Land’s Cycle only, naming those with statistically significant changes, highlighting the 5.37-fold upregulation of LPCAT4 upon differentiation (Benjamini-Hochberg corrected *p* = 4.76 × 10^−6^). (**E**) RT-qPCR timecourse of *LPCAT4* expression upon differentiation [D] with values normalised to the undifferentiated [UD] 0 h levels. Error bars represent standard deviation from three technical replicates of a single NHU donor background.

Urothelial differentiation elicits broad transcriptomic changes, reflecting the major shift from an actively proliferating cell monolayer to a transitional, stratified and quiescent epithelium with a tight electrophysiological barrier, as previously reported [19]. Strikingly, genes involved in lipid regulation and metabolism were highly enriched within the significantly changing genes (30.89% of expressed lipid regulatory genes; 23.26% of all expressed genes; *p* = 1.39 × 10^−7^) (Figure 2A). The undifferentiated state shows differential expression of genes involved in lipid biogenesis, including regulation of mitochondrial transport and fatty acid oxidation (Figure 2A,B). Differentiated urothelium switches from lipid biosynthesis to catabolic processes and lipid remodelling, including a striking example of the “sphingolipid rheostat” model [20], where both ceramide and the proliferation- and migration-inducing sphingosine-1-phosphate are remodelled in favour of the quiescence- and differentiation-associated sphingosine (Figure 2C). Differentiation also induced a sphingosine kinase switch from the pro-survival isoform 1, to the growth-inhibiting isoform 2; the latter at lower resting expression (Figure 2A,C). This is fully consistent with the well-described transcriptomic pattern of the sphingolipid rheostat associated with reduced sphingosine-1-phosphate abundance [21].

Given the dominance of PC in urothelial membranes (Figure 1A,B), the greater than 5-fold expression increase in the LPC-dominant acyl-CoA transferase *Lysophospholipid acyltransferase 4* (*LPCAT4*; previously known as *AGPAT7* [22] and *LPEAT2* [23], and recently proposed for a change of nomenclature to *LPLAT10* [24]) upon differentiation was striking (Figure 2D). Interrogation of the differentiation time course by RT-qPCR showed that *LPCAT4* was elevated immediately upon differentiation and then sustained (Figure 2E). Upregulation of the LPI-specific Membrane Bound O-Acyltransferase Domain Containing 7 (*MBOAT7*) did not contribute to any significant PI species by LC-MS/MS, suggesting an alternative role to membrane composition, likely in secondary signalling. Consistent with the LC-MS/MS profiling (Figure 1B), there were few changes across the glycerophospholipid de novo synthesis “Kennedy” pathway upon differentiation (Figure 2D).

### 2.3. LPCAT4 Knockdown Reduces Growth Rate but Elevates Urothelial Barrier Resistance

**Figure 3:**
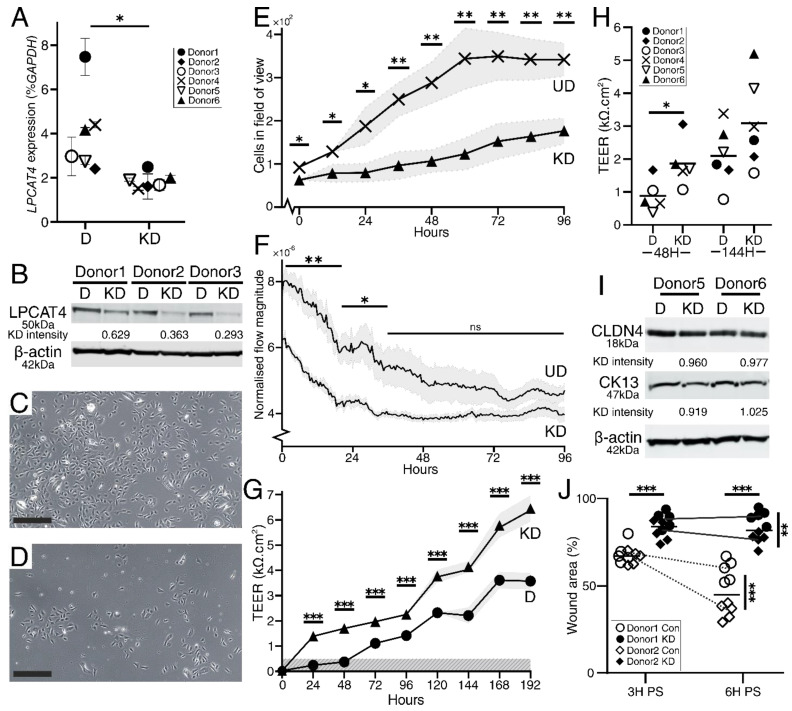
Characterisation of the *LPCAT4* knockdown [KD] cell lines compared to control undifferentiated [UD] or differentiated [D] cells. Donors exhibited variation in *LPCAT4* native expression as well as knockdown efficiency (**A**), but transcript-level knockdown was observed in all lines to an average of 50%. Similar knockdown was observed at the protein level (**B**). Transduced control cells (**C**) grew well, but matched LPCAT4 knockdown cultures (**D**) showed reduced population size after 9 days under transgene-selective conditions. Scale bars equal 200 μm. Reduced proliferation (**E**) and motility (normalised velocity assessed by optical flow) (**F**) were confirmed quantitatively using timelapse imaging. A significant difference in population size was even observed following the overnight acclimatising period from identical seeding densities, before recording began (0 h) (**E**). *LPCAT4* knockdown cultures consistently generated elevated ‘tighter’ electrophysical epithelial barriers (**G**). Again, donor NHU backgrounds exhibited barrier variance with and without knockdown, but the barrier was significantly elevated across all six donors at both early (48 h) and late (144 h) differentiation time points (**H**). Knockdown did not appear to impact NHU differentiation on western blots for either early (CK13) or later (CLDN4) markers (**I**). *LPCAT4* knockdown resulted in slower wound restitution after scratch wounding (**J**), but all wounds were eventually resolved. In (**A**), error bars for each donor represent the 95% confidence interval from all combinations of *GAPDH* and *LPCAT4* technical replicates. In (**E**–**G**) shaded regions represent the standard deviation range around the mean line, with time-matched pairwise statistical significance indicated. *** is *p* < 0.001, ** is *p* < 0.01, and * is *p* < 0.05 across all plots. In (**F**), measurements were taken every 30 min, so statistical significance is represented by regions of significance based on the average *p* value from a 3-comparison sliding window. Wound area and closure rate was assessed using the Hoechst 33258 images, with five technical repeats by random field selection.

We further investigated the role of *LPCAT4* in NHU cytodifferentiation, formation of an epithelial barrier and wound repair by establishing stable shRNA knockdowns in 6 independent NHU donor cell lines. Control and *LPCAT4* knockdown shRNA cultures were differentiated before assessing *LPCAT4* expression by RT-qPCR. *LPCAT4* expression was donor-dependent (Figure 3A), but was knocked down to an average of 50.88% across six independent cell lines (SEM 4.71; range 33.67–66.15). LPCAT4 protein knockdown was evaluated by Western blot and densitometry analysis in three donors, with a congruent average knockdown of 57.16% (Figure 3B).

Initial observations of the shRNA *LPCAT4* and shRNA control cultures suggested that growth rate was affected in the *LPCAT4* knockdown cells (Figure 3C,D). Quantitative analysis of undifferentiated shRNA-transduced NHU cell cultures using the Phasefocus LiveCyte™ showed significant inhibition of population size at each measured time point following seeding at identical densities (Figure 3E). Control shRNA cells exhibited a linear growth phase up to 60 h, when they became confluent. Comparison of the growth rates across this first 60 h period revealed a doubling time of 22.3 h in the control shRNA cells compared to 60.3 h in the *LPCAT4* knockdown cells. Similarly, knockdown cells exhibited significantly reduced motility compared to time-matched and seeding-density-normalised control cultures (Figure 3F). In both conditions, culture motility reduced as cells approached confluence (but at a non-significantly different rate; 0–21 h; *p* = 0.62). Resting normalised culture optical flow velocity at confluence appeared slightly reduced in the control, but non-significantly. Furthermore, there was no evidence of elevated cell death or sloughing in the knockdown cultures, supporting truly reduced growth and motility rates following *LPCAT4* knockdown.

Once induced to differentiate, formation of a tight epithelial barrier was determined by measuring transepithelial electrical resistance (TEER). Both control and knockdown shRNA cultures formed tight barriers (≥500 Ω.cm^2^), but *LPCAT4* knockdown generated a tight barrier more quickly, and sustained a significantly elevated barrier throughout the time course (maximal in both conditions at 192 h; Figure 3G). Absolute TEER values were donor-dependent, but were elevated in the *LPCAT4* knockdown lines (Figure 3H). Control and *LPCAT4* shRNA cultures showed no significant differences in early (CK13) or late (CLDN4) differentiation markers at the protein level (Figure 3I).

We investigated whether knockdown influenced urothelial repair after wounding, using a scratch-wound model of stratified cultures on glass in 2 independent donor backgrounds. Rate of wound restitution was donor-dependent, but typically wounds began to repair from 3 h and were resolved by approximately 10 h post-wound (representative images in Figure S1). *LPCAT4* knockdown significantly impaired scratch wound healing (*p* = 3.45 × 10^−6^ at 3 h and *p* = 5.00 × 10^−7^ at 6 h post-wound), with a highly donor-dependent closure rate (Figure 3J). As all wounds were eventually resolved, *LPCAT4* knockdown elicited a delayed response, rather than compromising the tissue entirely.

### 2.4. LPCAT4 Knockdown Reduces 18:1 Fatty Acyl Incorporation into Phosphatidylcholine

**Figure 4:**
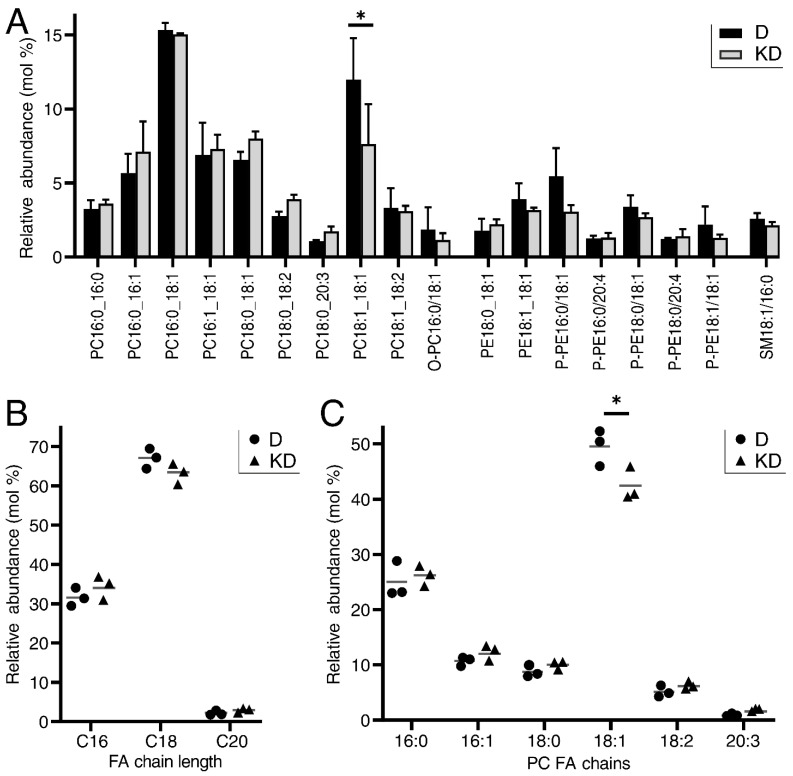
Impact of *LPCAT4* knockdown [KD] on the lipidomic profile of in vitro differentiated [D] human urothelial cells. (**A**) LC-MS/MS negative ion profile of lipid species with an average proportion ≥1% in either condition, highlighting the only significant difference in PC 18:1_18:1 molecules. Bars represent average relative proportion across 3 NHU transduced donor backgrounds (donors 1, 3 and 4; Figure 3) with statistical comparisons based on paired post hoc Tukey’s tests from an original 2-way ANOVA with BH-corrected significance values. (**B**) Glycerophospholipid fatty acyl chain length does not show significant differences upon knockdown, but monounsaturated C18 use in PC is significantly reduced upon *LPCAT4* knockdown (**C**). In (**B**,**C**), grey horizontal lines represent the average of 3 independent NHU donor backgrounds. Significance values were generated by paired ‘*t*’ tests, with * indicating *p* < 0.05.

We assessed the impact of *LPCAT4* knockdown on the glycerophospholipid environment by performing LC-MS/MS on differentiated shRNA control and knockdown cell lines from three donor backgrounds, utilising the same donors reported in Figure 1. Analysis focused on the negative ion channel where PC, PE and SM were represented (Figure 4A). Across all detected lipids, the only significant difference was a decrease in PC 18:1_18:1 molecules in *LPCAT4* knockdown cells (*p* = 0.013), which was also observed in the positive channel (*p* = 0.04; Supplementary Table S1). Overall PC proportion did not change, with averages of 58.1% in controls, and 58.0% in the knockdown. LPCAT4 has a known preference for 18:1 CoA [25], and concordantly the impact on 18:1 CoA acylation of LPC was only reliably observed in overall reduction of 18:1_18:1 PC. LPCAT4 preference for PC over PE was also observed, with no significant difference in PE 18:1_18:1 (*p* = 0.348) or P-PE 18:1_18:1 (*p* = 0.253). Slight, non-significant changes were observed in overall fatty acyl chain length usage across glycerophospholipids upon knockdown (Figure 4B), with the only significant difference a reduction in 18:1 usage in PC molecules (Figure 4C). Intriguingly, similar PC composition differences were not observed upon differentiation (Figure 1B), when *LPCAT4* gene expression (Figure 2C and Figure 3A) and protein abundance (Figure 3B) were significantly lower in the undifferentiated state.

### 2.5. LPCAT4 Knockdown Elicits Specific Transcriptomic Changes in Lipid Regulation, Cell-Cell and Cell-Matrix Interactions

**Figure 5:**
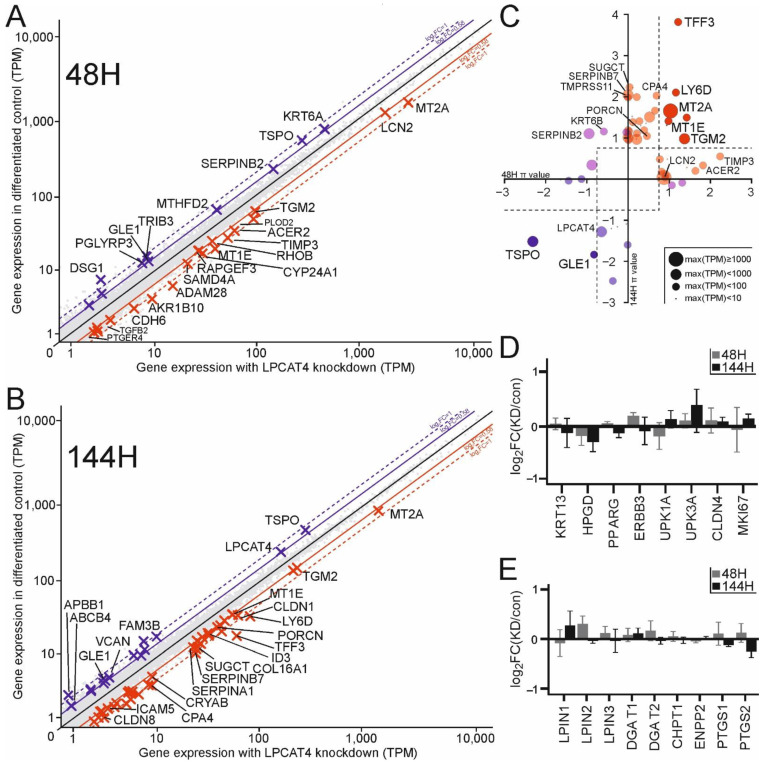
Specific transcriptomic effects of *LPCAT4* knockdown. Gene expression plot for 4 donor-matched knockdown and control cultures 48H (**A**) and 144H (**B**) after initiation of the NHU differentiation protocol. Genes upregulated following *LPCAT4* knockdown are indicated by red crosses and named when expression increases by at least 50% with a corrected *p* value less than 0.05 (two targets with smaller font are of interest have corrected *p* values less than 0.10). Genes downregulated by at least 50% with a corrected *p* value less than 0.05 are marked by blue crosses. Consistent transcriptomic changes between time points are indicated in (**C**) using π scores and scaled points based on maximum expression values. Bright red and blue indicate significant changes at both time points, transparencies represent significance at one time point. Pink circles represent genes where the fold-change direction switched between time points. There were no significant differences in differentiation markers at either time point (**D**), or in genes of lipid modifiers perhaps impacted by the reduction in LPCAT4 metabolic activity (**E**). Transduced donor backgrounds 1–4 (Figure 3 and Figure 4) were used for transcriptomic analysis.

LPCAT4 is not a transcription factor, and as an ER-bound protein it has limited potential for direct influence upon the cellular transcriptome. However, we investigated the overall impact of the altered lipid environment induced by *LPCAT4* knockdown on the transcriptome by performing mRNAseq at an early and late differentiation time point in four donor backgrounds (Table S2). Generally, changes to the global transcriptome were limited and typically exhibited low fold change, but specific genes relevant to the knockdown phenotypes described above were identified. Early in the differentiation protocol (Figure 5A) the changes between control and knockdown states were less numerous than at the later time point (Figure 5B). Whilst some of the identified genes were not modulated at both time points (Figure 5C), the same key processes were represented: cell-cell and cell-matrix interactions, matrix metallopeptidase activity, and regulation of lipid transport related to maintenance of the acyl-CoA pool. Markers of mature urothelial differentiation were not affected (Figure 5D).

Tight junction component genes *CLDN1* and *CLDN8* (a specific recruiter for CLDN4 [26]), as well as the adherens junction component *CDH6*, were upregulated in the knockdown cultures, while desmoglein *DSG1* was initially reduced. *LPCAT4* knockdown cells appeared less primed for matrix degradation with increased expression of the matrix metallopeptidase inhibitor *TIMP3* as well as the SERPIN family genes *SERPINA1*, *SERPINB7* and *SERPINB2* (latter switches fold change between 48 and 144 h), which actively inhibit wound healing driven by plasmin and TGF-β signalling in urothelium [27]. Upregulation of the metal ion regulating *MT2A* and *MT1E* also supported the modified activation state of metallopeptidases (e.g., *CPA4*, *CPZ* and *ADAM28*) upon knockdown. Furthermore, cell-matrix cross-linking genes were typically upregulated in the knockdown, including *PLOD2*, *RHOB*, *ICAM5*, *LCN2*, *COL16A1*, *TFF3* and *TGM2* (transglutaminase 2; TG2). Previous work found an increased expression of matrix-degrading inhibitors by NHU cells grown on Matrigel matrix, which was also associated with reduced migratory phenotype [28].

Interestingly, the changing dynamics of the acyl-CoA pool also impacted its broader regulation, with decreased *TSPO* transcript at both time points suggesting reduced efflux into the mitochondria [29] and differential stabilisation of the acetyl-CoA to acyl-CoA ratio to increase acyl-CoA (reduced acetyl > acyl activity via *MTHFD2* reduction, and increased acetyl > acyl via *AKR1B10*). Despite these broad CoA modulators, there were no specific changes in Lands’ Cycle or Kennedy Pathway genes upon *LPCAT4* knockdown (Figure 5E).

### 2.6. LPCAT4 Knockdown Phenotypes Are Recapitulated by Independent Inhibition of TSPO and PKC

Based on the *LPCAT4* knockdown transcriptomics, we identified two candidate, potentially impacted signal transduction pathways which might direct the observed changes in urothelial biology. We went on to investigate whether specific modulation of either of these pathways would phenocopy the effects of *LPCAT4* knockdown in NHU cells.

**Figure 6:**
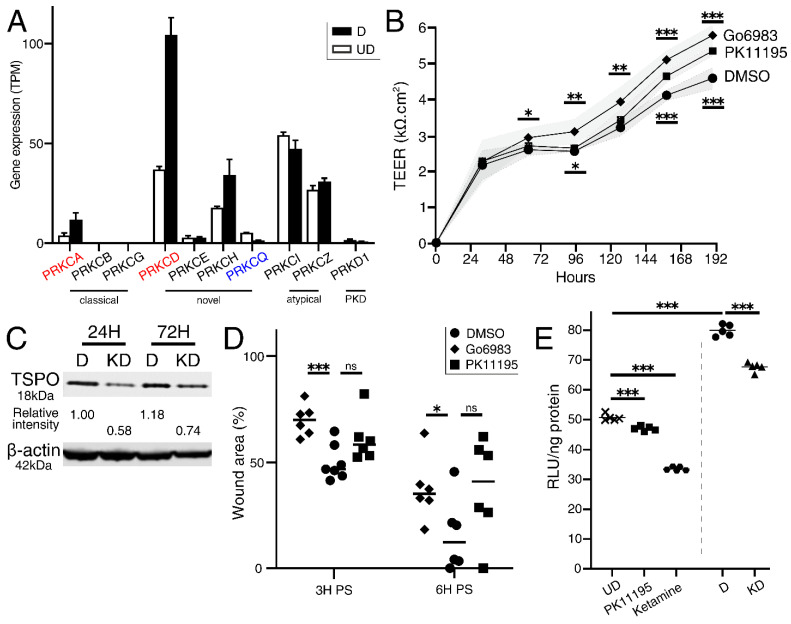
Phenocopying of the *LPCAT4* knockdown by independent inhibition of PKC and TSPO activity. (**A**) Expression of *PRKCD* is most abundant PKC gene in differentiated NHU cells, and significantly (by fold change and statistically) upregulated upon differentiation (red text; blue text for downregulated upon differentiation; data as described in Figure 2A). Classical PKC isozymes (DAG- and calcium-dependent) are less abundant. PKD (historically PKC-μ) shown for reference. (**B**) Inhibition of eitherpan-PKC (Go6983; significance indicated above trend lines) or TSPO (PK11195; significance indicated below trend lines) activity led to elevated urothelial barrier resistance. TEER values based on 6 technical replicates per condition per time point, with standard deviation represented as shaded areas around mean values. TSPO protein reduction in an *LPCAT4* knockdown environment was validated by western blotting (**C**) at 24 and 72-h after differentiation induction. Inhibition of PKC delayed wound healing (**D**), but high variance was observed with PK11195-treated cultures. (**E**) In proliferative conditions (left of dashed line), PK11195 treatment reduced NHU endogenous ATP availability by 6.9% (*p* = 1.82 × 10^−4^), with ketamine a strong positive control, as reported previously [33]. Upon differentiation (right of dashed line) endogenous ATP availability increased by 57.7% (*p* = 2.00 × 10^−9^). *LPCAT4* knockdown significantly reduced ATP availability by 15.7% (*p* = 3.82 × 10^−6^). Endogenous ATP fluorescence values from 5 technical replicates normalised by background blank cultures and total ng of protein. Across the figure, significance values were generated by independent ‘*t*’ tests, with * indicating *p* < 0.05, ** indicating *p* < 0.01, and *** indicating *p* < 0.001.

*TGM2* transcript was significantly increased in *LPCAT4* knockdown cells (Figure 5C). As TG2 protein directly inhibits Phospholipase C delta 1 (PLCδ1) hydrolysis of the PI derivative PIP2 to IP3 and DAG [30], we hypothesised that limited availability of DAG would reduce protein kinase C (PKC) activity. PKC-δ (*PRKCD*) was the most abundant family member in NHU cells, is differentiation-associated (Figure 2A and Figure 6A), and is DAG-dependent whilst independent of calcium [31]. We examined the effect of reduced PKC activity on urothelial physiology using Go6983, a well-characterised pan-PKC inhibitor [32]. Consistent with *LPCAT4* knockdown, PKC inhibition resulted in both a tighter urothelial barrier resistance (Figure 6B) and a significant delay to wound healing (Figure 6D).

TSPO was the most significantly downregulated transcript after *LPCAT4* knockdown (Figure 5C), and we confirmed TSPO protein was also reduced by Western blotting (Figure 6C). Furthermore, consistent with the *LPCAT4* knockdown, TSPO inhibition with PK11195 [34] led to a significant elevation of urothelial barrier resistance (Figure 6B). Impact on wound healing was non-significant (Figure 6D), but there was high variability between the cultures. TSPO has a broad role in cellular bioenergetics, including directly influencing ATP availability [34,35]. An ATP assay demonstrated significantly reduced ATP in cells treated with increased concentrations of PK11195 (Figure 6E), and further established that differentiated *LPCAT4* knockdown cells also contained significantly lower concentrations of endogenous ATP (Figure 6E).

## 3. Discussion

The master regulator of the urothelial differentiation programme is PPARγ [10], yet despite its parallel role in fatty acid regulation in adipogenesis [11], the regulation and modification of lipids in normal human urothelial differentiation is not fully understood. Here, LC-MS/MS profiling revealed large increases in TG abundance upon differentiation. TG biosynthesis genes were not upregulated transcriptomically, supporting a change of metabolism between proliferative and quiescent states. The corollary of this phenomenon is well known in epithelial cancers, including urothelial carcinoma [15,36]. Genes involved in lipid biosynthesis, regulation and signalling were enriched in genes differentially expressed between urothelial differentiation states, with an overall switch from biosynthesis to remodelling upon differentiation. Consistently, LC-MS/MS revealed no significant differentiation-associated changes in glycerophospholipid or sphingolipid abundance, however the transcriptomics supported the “sphingolipid rheostat” model [20] switch towards sphingosine from both ceramide and sphingosine-1-phosphate, as well as PC and PI remodelling through the Lands’ Cycle acyl transferases *LPCAT4* and *MBOAT7*. Whilst PI abundance across diverse mammalian membranes is lower than both PC and PE [2], its absence from the LC-MS/MS data supports a key role for phosphoinositide signalling in urothelial differentiation. PC is the most abundant glycerophospholipid in urothelial membranes, so the 5-fold increase in *LPCAT4* expression upon differentiation warranted further study.

Whilst differentiation-associated, our c.50% knockdown of *LPCAT4* transcript and protein in six independent biological backgrounds did not impact early and late markers of urothelial differentiation at either transcript or protein levels. However, *LPCAT4* knockdown did result in a significantly tighter epithelial barrier. Whilst barrier function is typically a marker of healthy urothelium [19], *LPCAT4* knockdown also resulted in delayed wound restitution, potentially driven by cell–cell and cell-matrix changes, particularly the enhanced expression of metalloproteinases known to inhibit classical plasmin-driven epithelial wound repair [37,38]. LC-MS/MS of the differentiated knockdown lines identified a specific reduction in 18:1 fatty acyls in PC; a previously reported LPCAT4 preference [25] not detected in a study of LPCAT4 activity in chondrocyte differentiation [39]. Pertinently, 18:1 abundance did not increase upon differentiation, even though *LPCAT4* abundance is much lower in the undifferentiated state, suggesting expanded glycerophospholipid diversity is more important in the fully differentiated quiescent tissue.

Whilst membrane PC changes in *LPCAT4* knockdown lines were specific, the magnitude of change was considered unlikely to have caused the broader impacts on urothelial tissue biology, specifically the increased barrier tightness and inhibited wound repair. We therefore examined a role for altered lipid signalling dynamics when LPC reacylation is reduced, although the specific mechanism remains unknown. Transcriptomic analysis of the *LPCAT4* knockdown suggested PLCδ1-driven hydrolysis of PIP_2_ may be impaired, reducing DAG availability for PKC activation. Previous epithelial studies have highlighted the role of PKC in regulating the activity of metallopeptidases and other cell-matrix regulators [40,41], as well as regulating tight junction assembly and dissolution [32]. Consistent with the *LPCAT4* knockdown phenotype, and these previous studies, direct inhibition of PKC activity in NHU cells elevated barrier tightness and reduced the rate of wound repair. The importance of DAG-reliant signalling in urothelial barrier integrity is further supported in the present study by identifying the upregulation of LPI-reacylator *MBOAT7* upon differentiation, the absence of high-abundance/low-diversity PI species in urothelial membranes, and a Kennedy Pathway phosphatidate phosphatase (PA→DAG) switch from *LPIN1* to *LPIN2/3* upon differentiation (known activators of *PPARG* expression in adipogenesis [42]). Our data support a role for elevated LPC concentrations in disrupting this key urothelial signalling pathway.

*LPCAT4* knockdown also resulted in downregulation of TSPO at the transcript and protein level. Again, the direct regulatory mechanism is unknown, although, pertinently, PKC-ε has been implicated in steroidogenic cells [43]. Whilst the impact of pharmacological TSPO inhibition on barrier tightness and wound healing was less striking (yet still consistent with *LPCAT4* knockdown), it highlighted reductions in endogenous ATP availability observed strongly in the *LPCAT4* knockdown environment, consistent with reduced proliferation rate and delays to urothelial wound healing, as described previously [44]. Taken together, these data support a key role for elevated LPC in modifying lipid signalling pathway outcomes in human urothelium.

In summary, we have performed the first paired lipidomic and transcriptomic characterisation of normal human urothelium, highlighting the importance of lipid mediators in the regulation of both urothelial differentiation and tissue homeostasis. The purpose of glycerophospholipid diversity in urothelium is yet to be fully elucidated. Knockdown of the differentiation-associated *LPCAT4* resulted in a tighter epithelial barrier but limited wound healing, consistent with LPC-driven changes to lipid signalling affecting both PKC activity and endogenous ATP availability. Barrier integrity and wound restitution are commonly compromised in both benign and malignant bladder disease, emphasising the need for a more complete characterisation of barrier regulation beyond protein localisation.

## 4. Materials and Methods

### 4.1. Tissues and Ethical Approval

NHS Research Ethics Committee approval was granted for the collection of human ureters from discarded tissue following renal transplant surgery from patients with no history of urothelial malignancy. The study was approved by The University of York Department of Biology Research Ethics Committee.

### 4.2. Normal Human Urothelial (NHU) Cell Culture and In Vitro Differentiation

Finite NHU cell lines were maintained in keratinocyte serum-free medium (KSFM; Invitrogen Europe Ltd., Inchinnan, UK) supplemented with recombinant epidermal growth factor, bovine pituitary extract and 30 ng/mL cholera toxin (Merck UK, Poole, UK), to derive KSFM “complete” (KSFMc). Under these conditions, NHU cells grow as an undifferentiated monolayer and maintain a proliferative phenotype. As previously described [19], NHU cells were induced to differentiate and form a biomimetic tissue with a physiological “tight” epithelial barrier [45] by culturing the cells in KSFMc supplemented with 5% adult bovine serum (ABS) for 3–4 days, then seeding 3–6 replicate 1.13 cm^2^ ThinCert^®^ membranes (Greiner Bio-One Ltd., Stonehouse, UK) at 5 × 10^5^ cells/membrane, cultured for a further 7 days in KSFMc with 5% ABS and [Ca^2+^] elevated to 2 mM. TEER was measured with the EVOM™ Voltohmmeter (World Precision Instruments, Hitchin, UK), or assessed continuously after membrane seeding using the cellZscope2 (nanoAnalytics, distributed through Labtech, Heathfield, UK).

### 4.3. Transduction of NHU Cell Lines with shRNA Constructs

In anticipation of generating targeted gene knockdowns, a non-specific, control short hairpin RNA (shRNA) construct provided with the Clontech RNAi-Ready pSIREn-RetroQ vector kit (Takara Bio UK Ltd., London, UK) was used to provide an experimental model control to future knockdowns. This control shRNA construct, complete with restriction overhangs for directional cloning and a MluI restriction site, was ligated into the RNAi-Ready pSIREN-RetroQ vector. Following bacterial transformation using XL-1 Blue supercompetent cells (Agilent Technologies LDA UK Ltd., Stockport), ligation was verified by MluI restriction digest. PT67 retroviral packaging cells were transfected with the verified pSIREN-RetroQ vector, and retroviral particles were collected from the medium before being passed through low-binding 0.45 μm Tuffryn^®^ filters (VWR International Ltd., Lutterworth, UK). As previously described [46], the filtered medium containing retrovirus was applied to undifferentiated, actively proliferating NHU cells, and transduced cells were selected using 1 μg/mL puromycin. To evaluate the impact of transduction on NHU differentiation state, triplicate cultures from two independent NHU cell lines were either transduced with the control shRNA, treated with only a puromycin-selection mock control, or not manipulated. Cells from each culture condition were differentiated as described above and RT-qPCR for early and late differentiation markers, as well as TEER values, were highly congruent, suggesting limited non-specific effects.

### 4.4. Identification of Lipid Regulators Associated with Urothelial Differentiation

mRNA sequencing data from donor-matched, undifferentiated (PRJNA847878) and in vitro differentiated (PRJNA610264) NHU cells, previously generated by our group, were used to identify urothelial differentiation-associated genes involved in lipid biosynthesis and regulation. Reads were quality-checked using FastQC v0.11.7 [47] and trimmed to remove adapters and low quality read ends using trimmomatic v0.36 [48]. Reads were pseudoaligned to the Gencode v35 human transcriptome using kallisto v0.46.0 [49] and gene-level transcripts per million (TPM) expression values derived using tximport v1.14.0 [50]. Donor-aware differential expression analysis at each timepoint was conducted using the likelihood ratio test in Sleuth v0.30.0 [51]. Gene set enrichment analysis was performed using the prerank module of GSEApy v0.10.2 [52], a Python3 wrapper for the Broad Institute’s GSEA tool [53], and the MSigDB v7.2 hallmark and gene ontology collections, run with 1000 permutations.

### 4.5. Derivation of LPCAT4 Knockdown Cell Lines

LPCAT4 protein functional and transmembrane domain locations were identified using InterPro v85.0 [54] and used to educate knockdown strategy. shRNA oligonucleotides were designed against the shared exonic sequences of the *LPCAT4* (ENSG00000176454) protein-coding transcripts in Gencode v28, using DSIR (http://biodev.cea.fr/DSIR/DSIR.html, accessed 8 January 2018) [55] and InvivoGen siRNA Wizard™ v3.1 (https://www.invivogen.com/sirnawizard/index.php, accessed 8 January 2018), followed by addition of a hairpin loop, restriction overhangs for directional cloning, and a MluI restriction site. Three unique sequences were designed: LPCAT4_shRNA1: gatccGCACCTGTTCCAACAAGAATTCAAGAGATTCTTGTTGGAACAGGTGCTTTTTTACGCGT (within ENSE00001253149); LPCAT4_shRNA2: gatccGTAGGGAGCTTACCTGTGATTTTCAAGAGAAATCACAGGTAAGCTCCCTACTTTTTTACGCGTg (within ENSE00003524206); LPCAT4_shRNA3: gatccGAATGATCAGCCAGGAAGAGTTTCAAGAGAACTCTTCCTGGCTGATCATTCTTTTTTACGCGTg (within ENSE00003630257). Each construct was transfected into retroviral packaging cells as described above. Two independent sets of donor-matched NHU cell lines were generated for the three *LPCAT4* shRNA constructs, and the control shRNA construct, and differentiated as described above. The impact of transduction on *LPCAT4* expression was assessed, as well as the knockdown efficiency of each *LPCAT4* shRNA construct by RT-qPCR and western blotting, and the culture doubling time was measured. These initial studies showed *LPCAT4* expression was unaffected by control transduction, and that the LPCAT4_shRNA2 construct gave the most effective *LPCAT4* transcript knockdown. 6 pairs of donor-matched NHU cell lines transduced with either control shRNA or LPCAT4_shRNA2 constructs were then generated and used for all further analyses.

### 4.6. Assessment of Culture Proliferation Rate

Whilst actively proliferating, transduced NHU cells were regularly imaged to assess culture doubling time. Doubling rate was formally quantified using the Phasefocus LiveCyte™ instrument (Phase Focus Ltd., Sheffield, UK) housed in the University of York (York, UK) Bioscience Technology Facility. Transduced cells from two independent backgrounds were seeded at 10^4^ cells/cm^2^ in triplicate onto 24-well Corning™ Primaria™ plasticware (suppled by Thermo Fisher Scientific UK, Loughborough, UK), allowed to adhere and acclimatise overnight in KSFMc, with cells counted every 12 h for 96 h.

### 4.7. Assessment of Culture Migration Rate

Videos of cell cultures obtained from the Phasefocus LiveCyte™ instrument were further analysed to assess cell migration rate, measured by normalised optical flow. Cells were counted from image frame sequences at 30 min intervals using ImageJ v1.53c (https://www.afterdawn.com/software/desktop/image_editing/imagej.cfm, last accessed 3 October 2022). First, non-uniform background was removed using the “Sliding Parabvoloid” function with a rolling ball radius of 50 pixels, then images were converted to 8-bit with a threshold applied in the range 0–210. Small visual artefacts in cell cytoplasm were removed using the “Fill Holes” function and watershedding applied to separate partially overlapped cells. Finally, “Analyse Particles” was applied to count all particles with radius greater than 50 pixels.

Following this preprocessing, images were loaded into Matlab 2021b (Computer Vision Toolbox, Mathworks, Natick, MA, USA) and converted to greyscale. The Matlab Image Processing toolbox function “estimateFlow” was then used to apply the opticalFlowHS “Horn-Schunck” method of estimating flow velocity characteristics on a per pixel basis between subsequent frames in each series. Velocity matrices were processed to obtain pixel velocity magnitude and orientation, with frame-wide averages normalised by observed cell number.

### 4.8. RT-qPCR

RNA was extracted from cell cultures using Trizol™ (Invitrogen Europe Ltd.) and cDNA synthesis performed as previously described [9]. Quantification assays were performed on an ABI Prism Real-Time PCR System (Thermo Fisher Scientific UK) using SYBR-green™ PCR master mix and PCR primers for *LPCAT4*, *UPK2*, *KRT13* and *GAPDH*. *LPCAT4* PCR primers (5′-AGCAGGATACCAAGGGTTTGG; 5′-GCCAGACGAGTTAGCTCTTCCA) were designed on the Applied Biosystems Primer Express™ Software, and existing primers were used for the other gene targets [9]. All values were quantified using the ΔΔCt method, and normalised to endogenous *GAPDH* expression.

### 4.9. Immunoblotting

Whole cell lysates (20 µg) were resolved on 4–12% Bis-Tris gels (Thermo Fisher Scientific UK) and transferred to PVDF membranes using standard immunoblotting techniques. Membranes were incubated with each primary antibody (Table 1) for 16 h at 4 °C. Secondary antibodies used were goat anti-mouse LI-COR IgG IRDye^TM^ 680 (Molecular Probes supplied by Thermo Fisher Scientific UK) or goat anti-rabbit IgG DyLight 800 (Generon, Slough, UK). Antibody binding was detected on the Odyssey^TM^ Infra-red Imaging System (Li-Cor Biosciences, Cambridge, UK) and the densitometry normalized to β-actin (Table 1).

### 4.10. Estimating Repair Capacity by Scratch-Wound Restitution

Transduced urothelial cells were seeded onto 12-well Multispot slides (Hendley-Essex, Loughton, UK) at 3.5 × 10^4^ cells/well, allowed to attach, then maintained for 7 days in differentiated culture conditions as described above. After the cells were scratch-wounded with a pipette tip, replicate slides were fixed in methanol:acetone (*v*/*v*) before wounding, and then at 0 h, 3 h, 6 h, 10 h and 72 h post-wound. Hoechst 33258 (0.1 µg/mL; Merck UK) was used to visualise nuclei. Wound size at each timepoint was measured using ImageJ and normalised to the 0 h condition-average scratch size.

### 4.11. Lipid Profiling of Transduced Urothelial Cell Lines

Twin cultures from three ureteric NHU cell lines (transduced donor backgrounds 1, 3 and 4) transduced with the scrambled shRNA sequence were expanded in proliferative conditions. From each twin culture, an undifferentiated, proliferative state was sampled by harvesting cells at ~70–80% visual confluence. The remaining culture was grown to visual confluence and then differentiated using medium supplemented with 5% ABS and 2 mM [Ca^2+^] for a further six days on plastic before harvest. Differentiated donor-matched *LPCAT4* shRNA knockdown cultures were also generated in the same manner. Harvested cells were pelleted and submitted to the University of York Bioscience Technology Facility for analysis. Cell pellets were lyophilised using a Genevac EZ-2.3 Elite Centrifugal Evaporator (Biopharma Group, Winchester, UK) and prepared for analysis by LC-MS/MS by the Bioscience Technology Facility Metabolomics and Proteomics staff, as described previously [56]. Downstream data analysis was performed in R, using the Bioconductor package XCMS [57] and processed as previously described [56]. MS1 spectra were matched against the LipidMaps Structural Database [58], and matching MS2 spectra were searched against the Lipid Match [59] and Lipid Blast databases [60]. Features were quantified relative to spiked-in deuterated standards (Avanti Polar Lipids Inc. SPLASH^®^ Lipidomix^®^, Stratech 330707-AVL). Lipid spectra were corrected using null and culture media controls, and species with abundance ≥ 1% were manually annotated by evaluating database results against likely MS1 adducts and retention times in positive and negative ionisation modes, and inspection of MS2 daughter ions and neutral losses. PC, PE, SM and TG species abundances were quantified using the class-specific SPLASH^®^ Lipidomix^®^ standards. Lipid profiles were compared by 2-way ANOVA, significance values corrected using Benjamini-Hochberg, and pairwise comparisons assessed by Tukey’s test.

### 4.12. Transcriptomic Profiling of Transduced NHU Cell Lines

A single ureteric NHU cell line was used to generate a differentiation time course of *LPCAT4* expression by RT-qPCR. *LPCAT4* expression was upregulated 24 h after media supplementation with ABS, and remained elevated throughout, validating previous data from microarray [61]. This suggested *LPCAT4* knockdown could affect initial differentiation and progression, as well as the final establishment of the differentiated phenotype.

Twin cultures of four donor-matched (transduced donor backgrounds 1–4) scrambled shRNA control and *LPCAT4* shRNA knockdown cell lines were expanded in proliferative culture conditions as described above. To assess early effect on barrier formation, cells were seeded onto triplicate 12-well 1.13 cm^2^ ThinCert™ culture membranes in KSFMc, allowed to adhere overnight, then the media supplemented with 5% ABS and 2 mM [Ca^2+^]. Half of each twin culture was harvested after two days in supplemented media, and the other after six days. TEER was assessed by EVOM™ Voltohmmeter before harvest. Total RNA was extracted using Trizol™ and submitted to the Oxford Genomics Centre (Oxford, UK) for polyA library synthesis and mRNA sequencing using the Illumina NovaSeq6000 (Illumina, Cambridge, UK) instrument, generating 150 bp paired-end reads (deposited in PRJNA848077). Sequencing data was checked for quality, processed and analysed as described above (independently at both time points), though no read trimming was required.

### 4.13. Phenocopying Effects of LPCAT4 Knockdown by Inhibition of PKC and TSPO

NHU cells from 2 donors were cultured in triplicate and differentiated as described above. Cells from each culture were seeded onto 1.13 cm^2^ ThinCert^®^ membranes (6 technical replicates per culture) as described, supplementing the differentiating medium with either 100 nM [32] pan-Protein Kinase C (PKC) inhibitor Go6983 (Tocris Bioscience, Avon, UK), or 100 nM [34] Translocator Protein (TSPO) inhibitor PK11195 (Tocris). Cultures were monitored for TEER, restitution of barrier after scratch-wounding, and rate of wound closure as mentioned above.

### 4.14. Quantification of Cellular ATP

Actively proliferating NHU cells were seeded on plastic at 10^4^ cells/well, allowed to attach, and treated for 48 h with either 0.1% DMSO (control), the TSPO inhibitor PK11195 at 100 nM or 500 nM, or with 3 mM ketamine, previously shown to exhibit a 23.2% reduction in urothelial cellular ATP [33]. Cellular ATP was quantified using the CellTiter-Glo^®^ 2.0 assay (Promega UK Ltd., Southampton, UK) with 5 technical replicates per condition, and normalised using a bicinchoninic acid protein assay kit (Thermo Fisher Scientific UK). Cells were lysed in CellTiter-Glo^®^ 2.0 reagent and assayed for luciferase activity in a Clariostar luminescence plate reader (BMG Labtech Ltd., Aylesbury, UK). Donor-matched control shRNA and *LPCAT4* shRNA knockdown differentiated cultures were also assayed for cellular ATP concentration.

## Figures and Tables

**Table 1:** Primary antibodies used in Western blotting experiments.

Antigen	Supplier	Reference	Host Species	Concentration
LPCAT4	Stratech, Ely, UK.	LS-C749318	Rabbit	1:3000
Claudin 4	Invitrogen Europe Ltd.	3E2C1	Mouse	1:1000
CK13	OriGene supplied by Cambridge Bioscience, Cambridge, UK	IC7	Mouse	1:500
TSPO	Abcam, Cambridge, UK	EPR5384	Rabbit	1:10,000
β-actin	Sigma, Merck UK	A5441	Mouse	1:10,000

## Data Availability

RNA sequencing raw data from differentiated ureteric urothelium was previously published (PRJNA610264). Data from the donor-matched undifferentiated cultures is now available at PRJNA847878. RNA sequencing data from the LPCAT4 knockdown and matched control cultures is available at PRJNA848077.

## Supplementary Materials

The following supporting information can be downloaded at: https://www.mdpi.com/article/10.3390/ijms231911871/s1.
